# A new species of *Cotesia* Cameron (Hymenoptera, Braconidae, Microgastrinae) reared from the hickory horned devil, *Citheronia regalis*, and luna moth, *Actias luna*, in east Texas

**DOI:** 10.3897/zookeys.740.242226

**Published:** 2018-02-27

**Authors:** James B. Whitfield, Robert J. Nuelle Jr., Robert J. Nuelle III

**Affiliations:** 1 Department of Entomology, 320 Morrill Hall, University of Illinois, Urbana, IL 61801 USA; 2 Research Associate, Entomology, Sam Houston State Natural History Collections Huntsville, TX 77340 USA

**Keywords:** Lepidoptera, parasitism, Saturniidae

## Abstract

The braconid wasp parasitoid *Cotesia nuellorum* Whitfield, new species, is described from specimens reared from a caterpillar of the hickory horned devil, *Citheronia regalis* (F.), and from a caterpillar of the luna moth, *Actias luna* (L.), in eastern Texas. The species is diagnosed with respect to other species of *Cotesia* recorded from North American Saturniidae, and details of its biology are provided.

## Introduction

*Cotesia* Cameron is a common and diverse genus of microgastrine Braconidae that largely specializes in parasitizing exposed larvae of macrolepidopteran moths and butterflies (Whitfield et al. 2018). It is one of the larger genera of Microgastrinae in terms of currently described species worldwide; its highest species richness lies in temperate zones, and it is relatively ubiquitous in terrestrial habitats where caterpillars occur.

Currently, four species in this genus are recorded from saturniid caterpillars in North America (Marsh 1979; Tuskes et al. 1996) – *C. anisotae* (Muesebeck), *C. electrae* (Viereck), *C. hemileucae* (Riley) and *C. teleae* (Muesebeck). Tuskes et al. (1996) provide a table of these associations along with records of other Nearctic saturniid parasitoids.

Recently, two of the authors (RJNJr and RJNIII) collected a batch of larvae of the hickory horned devil, *Citheronia regalis* (F.), in eastern Texas, and one of these larvae yielded a brood of *Cotesia* wasps (see below). While *Cotesia teleae* has been previously recorded as a parasitoid of *C. regalis* especially in the northeastern U. S. where the moth is now relatively rare (in addition to its more usual host *Antheraea polyphemus* (Cramer)), the Texas material appears to belong to a new species, described below. It is possible that at least some previous records of *C. teleae* from *C. regalis* actually belong instead to the new species, but we have been unable to confirm this. The geographical location of the Texas record places it far from the northeastern U. S., near the southwestern limit of the range of *C. regalis*, so it is not surprising if the parasitoid community is different in this ecologically distinct area.

Subsequently, *Cotesia* specimens reared by Richard S. Peigler from larvae of the luna moth, *Actias luna*, from the same area were found to be apparently conspecific, and are also included in our definition of the new species.

Below, JBW describes the new species of *Cotesia*, diagnosing it versus other species of *Cotesia* known to attack North American saturniids, and RJNJr and RJNIII provide discussion concerning its discovery and field biology.

## Materials and methods

**Figures 1, 2.**
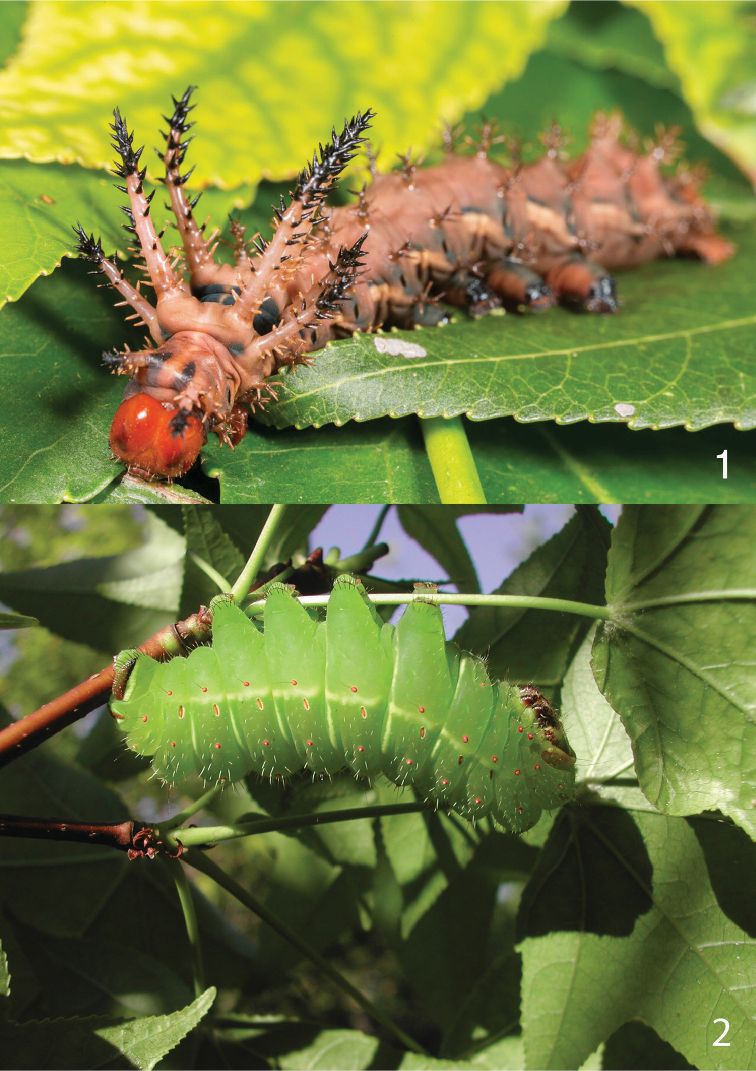
**1** Larva of the hickory horned devil, *Citheronia regalis* **2** Larva of the luna moth, *Actias luna*. Photo in **1** by Clayton Bownds, used with permission, photo in **2** by Richard S. Peigler, used with permission.

During October of 2014, RJNJr and RJNIII collected three caterpillars of *Citheronia regalis* (Fig. 1) in larval form on small American sweetgum (*Liquidambar styraciflua* L.) trees in the Sam Houston National Forest near Stubblefield Lake Park, Walker County, Texas. One of the specimens was a 2^nd^ or 3^rd^ instar caterpillar which subsequently died after about 13 days during the emergence of the braconid parasitoids described below. The parasitoid emergence was not observed by the authors, but the cocoons were saved and some were allowed to produce adult wasps. The original host, some larval parasitoids, cocoons, and adult parasitoids were saved for further study.

It was later noted that Richard S. Peigler had collected larvae of the luna moth, *Actias luna* (Fig. 2), in the same area, same month, but two years earlier, and recovered parasitoids that appeared similar in adult and cocoon appearance to those from *C. regalis*.

We examined these as part of the material described below.

**Figure 3.**
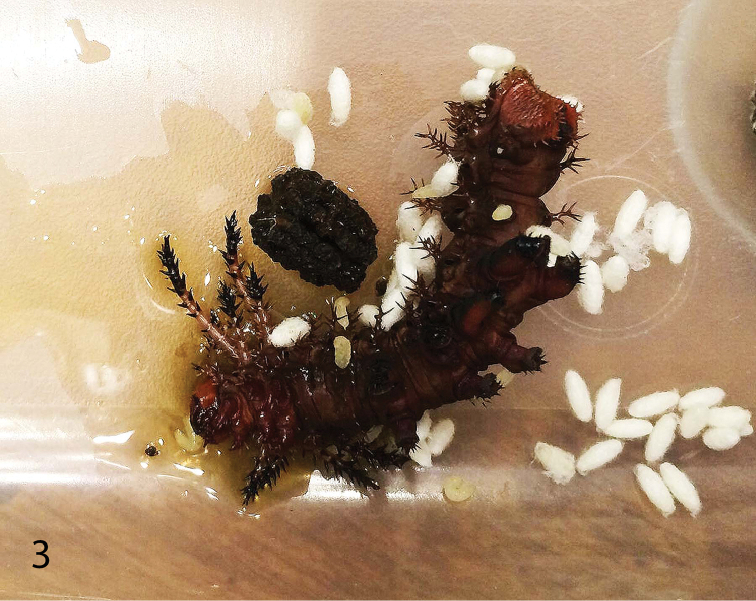
Larva of *C. regalis* with emerged larvae and cocoons of *C. nuellorum*, in rearing container. Photo by R. J. Nuelle, Jr.

The three caterpillars of *C. regalis* were raised on leaves of American sweetgum, *Liquidambar styraciflua* L., which were changed daily. The caterpillars were housed separately in well-ventilated plastic containers. The food plant was harvested daily, cleaned and the stems were trimmed under water to ensure a well-hydrated food source. Enclosures were cleaned daily. The caterpillars varied in size, with two appearing to be nearly mature larvae and the third appearing to be much younger. After 13 days, the smallest of the 3 caterpillars stopped eating, as if it was preparing to molt. The following morning the caterpillar was found lying on the floor of its enclosure (Fig. 3), surrounded by clear, luteous liquid, a large number of white, parasitoid cocoons and a few emerged wasp larvae.

The caterpillar host and 18 cocoons were immediately preserved together in 100 % ethyl alcohol. The other 30 cocoons were placed in a sealed container at room temperature for 5 days. During this period many of the wasps eclosed, and the sealed container was placed in the freezer for 3 days to kill all specimens. These specimens were placed in 100 % Ethyl alcohol.

The authors believe that this larva had been parasitized prior to capture, as none of the other larvae captured and reared with this specimen were likewise parasitized. One other more mature caterpillar, captured in the same area on the same day, completed its development successfully. It pupated 7 to 10 days after this parasitic incident occurred. A third specimen, captured in a different location, two weeks later, also completed its larval development and pupated successfully.

The reared *Cotesia* specimens from both *C. regalis* and from *A. luna* were compared to specimens of the described species of *Cotesia* known to attack Nearctic saturniid caterpillars (*C. anisotae* (Muesebeck), *C. electrae* (Viereck), *C. hemileucae* (Riley), *C. teleae* (Muesebeck)). All of these species were treated by Muesebeck in his (1920) revision of *Apanteles* (as then circumscribed), except *C. teleae*, which he described later (Muesebeck 1926). *C. teleae* in particular has been recorded to attack *Citheronia regal* is in the northeastern U.S., (Tuskes et al. 1996, see also Table 1), although it is most commonly recovered from the Polyphemus moth caterpillar, *A. polyphemus*. In both cases the parasitoids attack the earlier instars, and not the last instar larvae. The two species of *Cotesia* resemble each other in general appearance, but differ in the various features outlined below in the description. It remains to be seen whether *Cotesia* reared in other parts of North America from *C. regalis* are indeed *C. teleae* or sometimes belong to the new species described here. A molecular study of the complex of *Cotesia* species attacking Neartic saturniids is likely to reveal additional new species. The genus as a whole has proven taxonomically challenging except when ecological and/or molecular data are available to aid in species separation. A small table of described differences among the species attacking saturniids in North America is provided (Table 1), but there are no guarantees that the color characters listed will prove to be stable especially across broad geographic areas.

**Table 1.** Recorded hosts, cocoon types, and several color traits putatively varying between described species of *Cotesia* known to attack saturniid larvae in North America, including the new species described here. All of the species in this list are similar in having relatively smoother sculpturing on the propodeum and anterior metasomal tergites than is typical. *host are from Tuskes et al. (1996).

Cotesia species	Recorded hosts*	Cocoons	Tegulae	Fore and mid coxae	Hind femur
*anisotae*	*Anisota senatoria*	deep buff	blackish	black	black
	*A. stigma*	spun singly			
	*A virginiensis*				
	*Dryocampa rubicunda*				
*electrae*	*Agapema anona*	white	dark brown	variable	black
	*A. galbina*	spun singly			
	*A. homogena*				
	*Automeris io*				
	*Coloradia doris*				
	*C. pandora*				
	*Hemileuca burnsi*				
	*H. chinatiensis*				
	*H. eglanterina*				
	*H. electra*				
	*H. grotei*				
	*H. hera*				
	*H. nevadensis*				
	*H. oliviae*				
	*H. tricolor*				
*hemileucae*	*Automeris io*	white	yellowish	mostly yellowish	mostly yellowish
	*Hemileuca maia*	spun singly			
	*H. slosseri*				
*nuellorum*	*Citheronia regalis*	white	dark brown	mostly yellowish	mostly yellowish
	*Actias luna*	spun singly			
*teleae*	*Antheraea polyphemus*	white	yellowish	mostly black	variable
	*Citheronia regalis*	spun singly			

It is interesting that *Actias luna* is a commonly reared and widespread species that has not been officially recorded to yield *Cotesia* parasitoids before at any locality, although Peigler (1994) suggests that *C. teleae* might have been the species Fiske and Thompson (1909) found to attack earlier instar larvae of *A. luna* in experiments. The *Cotesia* from Peigler's rearing described here were tentatively previously identified as *C. schizurae* (Ashmead) (Peigler 2013), but that species has light buff-colored cocoons spun together in parallel rows, and attacks notodontids of the genus *Schizura*.

Possibly in nature this is an unusual association, and only occurred because *Actias* larvae co-occurred with *C. regalis* on sweetgum in this habitat. It remains to be seen if further rearings of the two host caterpillar species in east Texas continue to both yield *C. nuellorum*.

The description of the new *Cotesia* species presented below generally follows the terminology and format used in Fernandez-Triana et al. (2014) and uses primarily terms adopted by the Hymenoptera Anatomy Ontology (Yoder et al. 2010).

## Taxonomy

### Cotesia nuellorum

Taxon classificationAnimaliaHymenopteraBraconidae

Whitfield sp. n.

http://zoobank.org/1DEC4342-CBC6-444E-A0AF-6057B804C131

Figs 3 , 4–7

#### Type locality.

The original habitat is located within the Sam Houston National Forest, Walker County, Texas, near Stubblefield Lake Recreational area 338 feet AMSL (Lat: 30.524930 Lon: -95.622750 Accuracy: 10 m). This area is described as Pineywoods: Pine Forest or Plantation, according to the Texas Parks and Wildlife; Texas Ecosystem Analytical Mapper (TPWD T.E.A.M.) application. It is in a managed National Forest and is subject to occasional fire events. This successional area contains sweetgum, hickory, oak and various conifers as dominant trees. Many of the deciduous trees are relatively short (less than 6 feet tall) near the borders of roads and trails, and the generally open forest floor is thus highly convenient for sampling caterpillars.

#### Holotype.

Female deposited in USNM. TEXAS: Walker Co., Sam Houston National Forest, nr. Stubblefield Lake, 30.524930, -95.622750, October 2014, 100 m. elev., coll. R. J. Nuelle, Jr. and R. J. Nuelle, III, ex larva *Citheronia regalis* on sweetgum.

#### Paratypes.

4 females, 1 male with same data as holotype, plus 26 females, 7 males (deposited in CNC, INHS, SHSU, TAMUIC, UWIM (Laramie)) from: TEXAS: Walker Co., Sam Houston National Forest, Stubblefield Lake, ex. larva *Actias luna* on sweetgum, em. 21-22-X-2012, coll. R. S. Peigler.

#### Description.

**Female.** Body color: body mostly dark except palps, portions of legs (see below) and ventral portions of anterior laterotergites. Antenna color: scape black, pedicel dark brown, flagellum dark brown/black. Coxae color (pro- , meso, metacoxa): honey yellow; honey-yellow; black proximally, shading to medium brown distally. Femora color (pro-, meso-, metafemur): honey-yellow; honey-yellow; honey-yellow with smoky spot dorsally near distal end. Tibiae color (pro-, meso-, metatibia): honey-yellow; honey-yellow; honey-yellow with darkened extreme distal end. Tegula and humeral complex color: tegula dark brown translucent, humeral complex dark brown translucent (both slightly more translucent and paler in males). Pterostigma color: dark greyish brown, with indistinct paler junction with C+SC. Fore wing veins color: partially pigmented (a few veins may be dark but most are pale – see figure for pattern). Antenna length/body length: antenna approximately as long as body (head to apex of metasoma). Body in lateral view: not distinctly flattened dorso–ventrally. Body length (head to apex of metasoma): 2.0–2.2 mm. Fore wing length: 2.2–2.4 mm. Ocular–ocellar line/posterior ocellus diameter: 2.3–2.5. Interocellar distance/posterior ocellus diameter: 2.0–2.2. Antennal flagellomerus 2 length/width: 2.9–3.1. Antennal flagellomerus 14 length/width: 1.9–2.1. Length of flagellomerus 2/length of flagellomerus 14: 2.1–2.3. Metafemur length/width: 3.2–3.3. Metatibia inner spur length/metabasitarsus length: 0.4–0.5, about 10% longer than outer spur. Anteromesoscutum: mostly with shallow, dense punctures (separated by less than 2.0 × maximum diameter), but with polished and virtually punctureless strip near scutoscutellar sulcus. Mesoscutellar disc: mostly punctured but sometimes indistinctly so. Number of pits in scutoscutellar sulcus: 9 or 10. Propodeal carinae: strong medial longitudinal carina, vague hints of a transverse carina both otherwise rugose, especially medially and anteriorly. Mediotergite 1 length/width at posterior margin: 0.9–1.1. Mediotergite 1 shape: barrel-shaped, broadest in posterior 0.2. Mediotergite 1 sculpture: mostly sculptured, albeit shallowly, otherwise shiny, especially anteriorly. Mediotergite 2 width at posterior margin/length: 2.0–2.2. Mediotergite 2 sculpture: punctate/rugose, but shinier and smoother laterally. Outer margin of hypopygium: evenly sclerotized, posterior margin reaching tip of metasoma and forming a shallow even convex curve. Ovipositor thickness: evenly narrowing towards tip. Ovipositor sheaths exerted but visible portion shorter than hypopygium length. Length of fore wing veins r/2RS: 1.1–1.2. Length of fore wing veins 2RS/2M: 1.1–1.3. Length of fore wing veins 2M/(RS+M)b: 0.9–1.0. Pterostigma length/width: 3.1–3.5. Point of insertion of vein r in pterostigma: at roughly half way point length of pterostigma. Angle of vein r with fore wing anterior margin: nearly perpendicular, slightly inclined towards fore wing apex. Shape of junction of veins r and 2RS in fore wing: distinctly but not strongly angled.

**Male.** As female but with slightly darker legs, more polished tergites and sometimes more translucent and paler tegulae. Body size usually about 10 % smaller than female.

**Figures 4–7.**
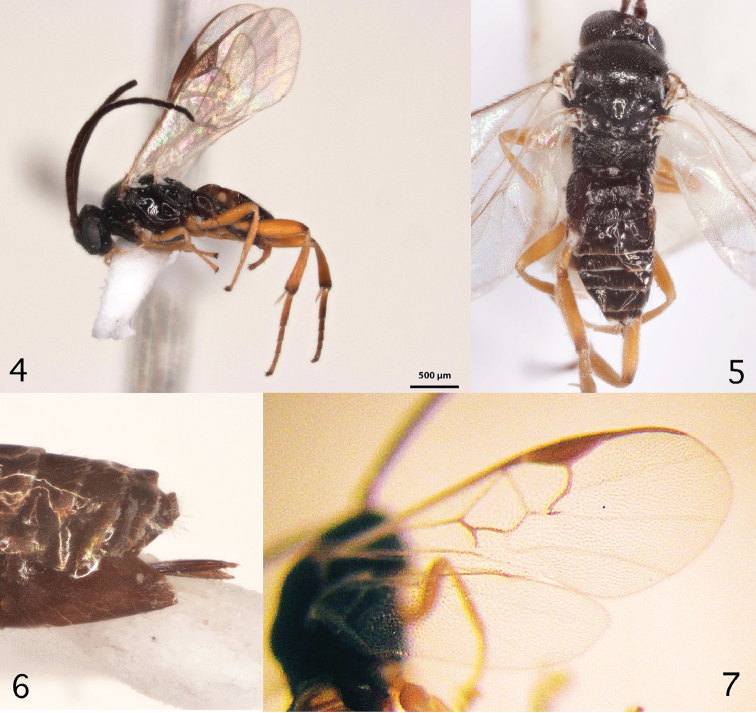
**4** Lateral habitus photo of *Cotesia nuellorum* female **5** Dorsal habitus photo of *Cotesia nuellorum* female **6** lateral view of posterior end of metasoma of *C. nuellorum*, showing hypopygium and ovipositor sheaths **7** Wings of *C. nuellorum* female.

#### Molecular data.

None yet recorded. A broad sample of *Cotesia* reared from various larger Nearctic saturniids would be useful to clarify how distinct the parasitoid species are both in terms of host specificity and in terms of geographic distribution. In Costa Rica, where the diversity of Saturniidae is higher, the host specificity, at least to host genus, appears high (Smith et al. 2008; Janzen and Hallwachs 2017).

#### Biology/ecology.

Gregarious (Fig. 3) on early instar larvae of host. Host: Saturniidae: Ceratocampinae: *Citheronia regalis* (F.) and Saturniinae: *Actias luna* (L.). 4^th^ and 5^th^ instar larvae do not appear to serve as hosts, as with some other *Cotesia* parasitizing large *Sphingidae* and *Saturniidae*.

#### Distribution.

Known so far only from Texas but likely to be much more widely distributed.

#### Ecologically and/or morphologically similar species.

Table 1 provides a comparison of the species so far known from saturniids in North America.

#### Etymology.

This species is named by JBW for the original discoverers, Robert J. Nuelle, Jr. and Robert J. Nuelle, III.

## Supplementary Material

XML Treatment for Cotesia nuellorum
